# Comparative Whole Genome Analysis of *Escherichia coli* O157:H7 Isolates From Feedlot Cattle to Identify Genotypes Associated With the Presence and Absence of *stx* Genes

**DOI:** 10.3389/fmicb.2021.647434

**Published:** 2021-04-01

**Authors:** Mo Jia, Ifigenia Geornaras, Jennifer N. Martin, Keith E. Belk, Hua Yang

**Affiliations:** Center for Meat Safety and Quality, Department of Animal Sciences, Colorado State University, Fort Collins, CO, United States

**Keywords:** whole genome analysis, Shiga toxin-producing and *stx*-negative *E. coli* O157:H7, cattle, acquisition and loss of *stx* genes, Red homologous recombination, IS629, metabolic pathway genes, evolutionary pathways

## Abstract

A comparative whole genome analysis was performed on three newly sequenced *Escherichia coli* O157:H7 strains with different *stx* profiles, previously isolated from feedlot cattle [C1-010 (*stx1−*, *stx2c*+), C1-057 (*stx−*), and C1-067 (*stx1*+, *stx2a*+)], as well as five foodborne outbreak strains and six *stx*-negative strains from NCBI. Phylogenomic analysis demonstrated that the *stx2c*-carrying C1-010 and *stx*-negative C1-057 strains were grouped with the six NCBI *stx*-negative *E. coli* O157:H7 strains in Cluster 1, whereas the *stx2a*-carrying C1-067 and five foodborne outbreak strains were clustered together in Cluster 2. Based on different clusters, we selected the three newly sequenced strains, one *stx2a*-carrying strain, and the six NCBI *stx*-negative strains and identify their prophages at the *stx* insertion sites. All *stx*-carrying prophages contained both the three Red recombination genes (*exo*, *bet*, *gam*) and their repressor *cI*. On the other hand, the majority of the *stx*-negative prophages carried only the three Red recombination genes, but their repressor *cI* was absent. In the absence of the repressor *cI*, the consistent expression of the Red recombination genes in prophages might result in more frequent gene exchanges, potentially increasing the probability of the acquisition of *stx* genes. We further investigated each of the 10 selected *E. coli* O157:H7 strains for their respective unique metabolic pathway genes. Seven unique metabolic pathway genes in the two *stx2a*-carrying strains and one in the single *stx2c*-carrying and seven *stx*-negative strains were found to be associated with an upstream insertion sequence 629 within a conserved region among these strains. The presence of more unique metabolic pathway genes in *stx2a*-carrying *E. coli* O157:H7 strains may potentially increase their competitiveness in complex environments, such as feedlot cattle. For the *stx2c*-carrying and *stx*-negative *E. coli* O157:H7 strains, the fact that they were grouped into the same phylogenomic cluster and had the same unique metabolic pathway genes suggested that they may also share closely related evolutionary pathways. As a consequence, gene exchange between them is more likely to occur. Results from this study could potentially serve as a basis to help develop strategies to reduce the prevalence of pathogenic *E. coli* O157:H7 in livestock and downstream food production environments.

## Introduction

Foodborne pathogenic *Escherichia coli* O157:H7 causes more than 96,000 cases of diarrheal illness and 3,200 hospitalizations annually in the United States (Scallan et al., 2011). This pathogen can cause severe gastrointestinal illness, including bloody diarrhea, which may progress to more serious illness, such as hemolytic uremic syndrome (HUS) and even death (Karmali, 2004). The *eae* (encoding intimin) and *stx* (encoding Shiga toxin) harbored in foodborne pathogenic *E. coli* O157:H7 strains are central to the pathogenesis of HUS (Paton and Paton, 1998). In addition to causing HUS, Shiga toxin produced by *E. coli* O157:H7 can enhance the adherence to epithelial cells and colonization in mice intestines (Robinson et al., 2006). Compared with Shiga toxin-producing *E. coli* O157:H7 that cause severe illness, *stx*-negative *E. coli* O157:H7 strains do not produce Shiga toxin. Though they may cause symptoms, such as diarrhea, they are not generally associated with HUS, even though they still carry virulence factors, such as *eae* and *bfpA* genes (Black et al., 2010; Ochoa and Contreras, 2011; Ferdous et al., 2015).

For decades, conventional molecular methods, such as polymerase chain reaction (PCR) and Sanger sequencing, have provided valuable information on the potential mechanisms of the acquisition or loss of the *stx* genes in the chromosome of *E. coli* O157:H7 cells. A previous PCR-based study found that *stx*-negative *E. coli* O157:H7 could acquire the *stx* genes in food animals (bovine and avian) and in feedlot environments by lysogenization of *stx*-carrying prophages into the *stx* insertion sites in bacterial genomes (Wetzel and LeJeune, 2007). Additionally, in humans, *stx*-positive *E. coli* O157:H7 may lose their *stx*-carrying prophages and, as a result, be converted into *stx*-negative *E. coli* O157:H7 during the infection process (Bielaszewska et al., 2007; Käppeli et al., 2011; Ferdous et al., 2015).

In addition to the *stx* genes, studies have also been conducted to compare the antimicrobial resistance of *stx*-positive and *stx*-negative *E. coli* O157:H7 isolates. A recent study compared antibiotic resistance genes in 19 *stx*-positive and 8 *stx*-negative *E. coli* O157:H7 isolates from cattle (Um et al., 2018). These *E. coli* O157:H7 isolates were screened, using a triplex real-time PCR assay, for 14 resistance genes including *bla*_*TEM*_, *strA–strB*, *addA1*, *tet(A)*, *tet(B)*, *sulI*, *sulII*, *sulIII*, *cmlA*, *catI*, *catII*, *catIII*, and *floR*. None of the eight *stx*-negative *E. coli* O157:H7 strains carried any antibiotic resistance genes, whereas one of the *stx*-positive strains contained several antibiotic resistance genes including *bla*_*TEM*_, *strA–strB*, *tet*(A), and *sulII*, and another *stx*-positive strain harbored a single *tet*(A) gene (Um et al., 2018). Another study compared the resistance to nine antibiotics in 12 *stx*-positive and 17 *stx*-negative *E. coli* O157:H7 isolates from bovine, caprine, and ovine milk in Greece using the disk diffusion method (Solomakos et al., 2009). The nine antibiotics evaluated included amoxicillin + clavulanic acid, ampicillin, cefachlor, cefuroxime, chloramphenicol, gentamicin, streptomycin, tetracycline, and trimethoprim + sulfamethoxazole. On average, the 12 *stx*-positive isolates were resistant to higher numbers of antibiotics (six of nine) than the 17 *stx*-negative isolates (four of nine) (Solomakos et al., 2009). These two studies suggest that the presence or absence of the *stx* genes between *stx*-positive and *stx*-negative *E. coli* O157:H7 may also result in changes in other phenotypic traits, such as antibiotic resistance.

The conventional molecular methods used to compare *stx*-positive and *stx*-negative *E. coli* O157:H7 are limited by their capabilities to study only certain selected DNA sequences from the bacterial genome, such as individual genes and/or short DNA fragments. With the development of next generation sequencing technology and whole genome sequencing (WGS), researchers are now able to compare and analyze DNA sequences at the level of the entire bacterial genome. A whole genome comparison study of *E. coli* O157 isolates from patients and cattle indicated that *stx*-negative *E. coli* O157:H7/NM isolates shared 22 virulence genes, the locus of enterocyte effacement region, and plasmids with *stx*-positive *E. coli* O157:H7 isolates (Ferdous et al., 2015). Another study used timed phylogeny analysis of whole genome sequences to investigate the evolution of a clinical *stx*-positive *E. coli* O157:H7 strain (Byrne et al., 2018). From this analysis, the authors reported that *stx2a*-carrying *E. coli* O157:H7 had evolved from *stx*-negative *E. coli* O157:H7 by acquiring a *stx2a*-carrying prophage, and that the *stx*-negative *E. coli* O157:H7 had evolved from a *stx2c*-carrying *E. coli* O157:H7 by losing a *stx2c*-carrying prophage (Byrne et al., 2018). In addition, a recent study used phylogenetic analysis to plot plasmids according to their phylogenetic positions in 14 *E. coli* O157:H7 and O157:NM strains that emerged stepwise (Nyong et al., 2020). By comparing *stx*-positive and *stx*-negative *E. coli* O157:H7 isolates from patients, cattle feces, and swine feces, they observed a stable evolutionary relationship between the host chromosomes and their respective plasmids. This previous study suggested the coevolution of plasmids and the chromosome in *E. coli* O157:H7/NM.

Cattle are one of the primary reservoirs of both *stx*-positive and *stx*-negative *E. coli* O157:H7 (Orden et al., 2002; Kuhnert et al., 2005; Keen et al., 2006; Sanchez et al., 2010). While pathogenic *stx*-positive *E. coli* O157:H7 are frequently associated with severe disease in humans, food animals are often asymptomatic carriers because they lack vascular Shiga toxin receptors (Pruimboom-Brees et al., 2000; Ferens and Hovde, 2011). Previous studies conducted to compare the whole genome sequences of *stx*-positive and *stx*-negative *E. coli* O157:H7 have mainly focused on human isolates. Similar such studies with *stx*-positive and *stx*-negative *E. coli* O157:H7 isolates of food animal origin are limited.

The current study was conducted to better understand the detailed mechanisms involved in the acquisition or loss of *stx* genes and the particular genotypes associated with the presence or absence of *stx* genes in *E. coli* O157:H7 isolated from feedlot cattle. The feedlot cattle *E. coli* O157:H7 strains included in the present study were from a previously conducted study (Carlson et al., 2009). The PCR results demonstrated that these *E. coli* O157:H7 strains displayed different *stx* profiles. The majority of the *E. coli* O157:H7 isolates from the study by Carlson et al. (2009) carried both or either *stx1* or *stx2* genes, but some of the *E. coli* O157:H7 isolates were *stx*-negative (i.e., neither *stx1* nor *stx2* genes present). In the present study, we conducted comparative genomic analysis of three isolates with different *stx* profiles to investigate their virulence genes, genes involved in the acquisition or loss of *stx* genes, and genes involved in metabolic pathways. Furthermore, the WGS data of these three strains were compared with those of five foodborne outbreak *E. coli* O157:H7 strains and six *stx*-negative *E. coli* O157:H7 strains from animal and food sources. Comparative whole genome analysis of *E. coli* O157:H7 strains with different *stx* gene profiles obtained from animal and food sources will expand our understanding of the mechanisms of exchange of *stx* genes and other genes associated with the exchange of *stx* genes between *stx*-positive and *stx*-negative *E. coli* O157:H7. Results from this study could potentially serve as a basis to help develop potential strategies to reduce the prevalence of pathogenic *E. coli* O157:H7 in livestock and downstream food production environments.

## Materials and Methods

### Strain Selection and Whole Genome Sequencing

Three *E. coli* O157:H7 strains with different *stx* profiles were selected for this study. These included strains C1-057 (*stx1−*, *stx2-*), C1-010 (*stx1−*, *stx2c*+), and C1-067 (*stx1*+, *stx2a*+), and all three strains were previously isolated from fecal samples collected from cattle in a commercial feedlot (Carlson et al., 2009). For the *stx*-negative *E. coli* O157:H7 strain C1-057, we have previously reported its draft genome sequence (NCBI accession no. LAZO01000000; Yang et al., 2016). Following publication of the draft genome sequence, further sequencing was conducted by an external laboratory (University of Washington), using the PacBio RSII system. A complete, gapless chromosome and a plasmid of this strain were deposited into NCBI with new NCBI accession number (chromosome CP035366.1; plasmid CP035367.1). Additionally, the two *stx*-positive *E. coli* O157:H7 strains, C1-010 and C1-067, were sequenced *via* the PacBio Sequel system. The two *stx*-positive *E. coli* O157:H7 strains were activated from frozen glycerol stocks (−80°C) by two transfers in tryptic soy broth (TSB; Difco, Becton Dickinson and Co., Sparks, MD, United States) at 37°C for 24 h. The genomic DNA of each isolate was extracted using a QIAGEN Genomic DNA kit (QIAGEN, Redwood City, CA, United States). For the PacBio platform, the extracted large genomic DNA needs to be fragmented into smaller size by *Eco*RI restriction enzyme prior to DNA sequencing. If *Eco*RI restriction recognition sites were methylated, the restriction enzyme would not be able to cleave the DNA. In our study, a small amount of extracted DNA sample was digested with *Eco*RI. Then, gel electrophoresis was used to prove that *Eco*RI restriction recognition sites were not methylated, and the genomic DNA could be fragmented into smaller size. The extracted DNA samples were sent out for sequencing service by Genewiz, Inc. (San Francisco, CA, United States).

The PacBio Sequel system was used to produce the raw reads with mean genome coverages of 477x for strain C1-010 (*stx1−*, *stx2c*+) and 497x for strain C1-067 (*stx1*+, *stx2a*+). The lengths of raw reads were between 3 and 40 kb. The raw reads of strains C1-010 and C1-067 were subsequently *de novo* assembled into contigs separately by Canu (version 1.6). When fully assembled, the genomes of strains C1-010 and C1-067 consisted of 13 and 12 scaffolds, respectively. To identify plasmids, DNA sequences of pO157 (NCBI accession no. NC_002128) and pOsak1 (NCBI accession no. AB011548) of *E. coli* O157:H7 strain Sakai were used as a reference for Basic Local Alignment Search Tool (BLAST) analyses (Altschul et al., 1990). Strain Sakai was used as it is a well-studied foodborne pathogenic *E. coli* O157:H7 strain that was responsible for a large outbreak in Japan (Hayashi et al., 2001). Chromosome and plasmid sequences of the three newly sequenced strains were annotated using the NCBI Prokaryotic Genome Annotation Pipeline (Tatusova et al., 2016). The sequences of strains C1-057, C1-010, and C1-067 were deposited in the NCBI database, and their NCBI accession numbers are listed in Table 1.

**TABLE 1:** The *stx* gene profiles, accession numbers, sources, and countries of origin of the *Escherichia coli* O157:H7 strains used in this study.

**Strains**	***stx* profile**	**NCBI accession no.**	**Source**	**Origin**	**Related cases**	**References**
**Newly sequenced *E. coli* O157:H7 strains**						
C1-010	*stx2c*	NZ_SCKH00000000	Cattle feces	United States	N/A	Carlson et al. (2009)
C1-057	*stx–*	CP035366.1	Cattle feces	United States	N/A	Carlson et al. (2009)
C1-067	*stx1*, *stx2a*	RICC01000000	Cattle feces	United States	N/A	Carlson et al. (2009)
**Foodborne outbreak *E. coli* O157:H7 strains**						
EC4115	*stx2a*, *stx2c*	NC_011353.1	Human	United States	2006 spinach outbreak	Eppinger et al. (2011)
TW14359	*stx2a*, *stx2c*	NC_013008.1	Human	United States	2006 spinach outbreak	Kulasekara et al. (2009)
Sakai	*stx1*, *stx2a*	NC_002695.2	Human	Japan	1996 outbreak	Hayashi et al. (2001)
EDL933	*stx1*, *stx2a*	CP008957.1	Ground beef	United States	1982 outbreak	Latif et al. (2014)
Xuzhou21	*stx1*, *stx2a*	NC_017906.1	Human	China	1999 outbreak	Xiong et al. (2012)
***stx*-negative *E. coli* O157:H7 strains**						
21B8	*stx–*	CP040309.1	Cattle	United States	N/A	National Center for Biotechnology Information (2019)
NZRM3614	*stx–*	CP032793.1	Unknown	Austria	N/A	National Center for Biotechnology Information (2019)
M7638	*stx–*	CP040313.1	Unknown	United States	N/A	National Center for Biotechnology Information (2019)
F1 E4	*stx–*	CP040307.1	Cattle	United States	N/A	National Center for Biotechnology Information (2019)
CV261	*stx–*	CP040316.1	Cattle	France	N/A	National Center for Biotechnology Information (2019)
MA11	*stx–*	CP040314.1	Meat	Malaysia	N/A	National Center for Biotechnology Information (2019)

In addition to the three newly sequenced *E. coli* O157:H7 strains, we also included five foodborne outbreak *E. coli* O157:H7 strains and six *stx*-negative *E. coli* O157:H7 strains obtained from the NCBI database (Table 1). These strains were selected to represent the different *stx* profiles, and their genomes were compared with the three newly sequenced strains. The *stx* gene profiles, origins, and NCBI accession numbers of all 14 *E. coli* O157:H7 strains used in this study are listed in Table 1.

### Phylogenomic Analysis

A phylogenomic analysis was performed on all 14 *E. coli* O157:H7 strains listed in Table 1. The phylogenomic tree was generated based on the single nucleotide polymorphisms (SNP) matrix between the *E. coli* O157:H7 strains using kSNP3.1 (available at^1^) (Gardner et al., 2015). The kSNP3.1 is a non-reference-based SNP analysis software suite that incorporates multiple SNP utility programs on a Linux platform. The parameter k-mer size was determined by Kchooser, a utility program incorporated in kSNP3.1. Chromosome FASTA files of the 14 strains obtained from the NCBI database were imputed in kSNP3.1 to generate the SNP matrix. The SNP matrix output was subsequently used to construct a maximum likelihood (ML) phylogenic tree by MEGA-X (available at^2^) with the parameter set at 2,000 bootstraps (Kumar et al., 2018).

### Identification of Prophages

Based on results from the phylogenomic analysis, 10 *E. coli* O157:H7 strains were selected for prophage identification. Phage Search Tool Enhanced Release (PHASTER) (available at^3^) is a web-based service for rapid identification and annotation of prophage sequences within bacterial genomes (Arndt et al., 2016). Prophages in the newly sequenced strains, C1-057 (*stx1−*, *stx2-*), C1-010 (*stx1−*, *stx2c*+), and C1-067 (*stx1*+, *stx2a*+), the reference strain Sakai (*stx1*+, *stx2a*+), and the six aforementioned *stx*-negative strains (Table 1) were identified by PHASTER. GenBank files of these strains were uploaded to the PHASTER server for identifications of intact, questionable, and incomplete prophages harbored in each of the strains. Strain Sakai was used as the *stx*-positive *E. coli* O157:H7 reference strain in this analysis since it is a well characterized foodborne pathogenic strain containing 18 prophages (Hayashi et al., 2001).

### Identification of the *stx* Insertion Sites and Red Recombination Genes

DNA sequences of six previously reported *stx* insertion sites (*wrbA*, *sbcB*, *yehV*, *argW*, *yecE*, and *Z2577*) were extracted from the whole genome sequence of *E. coli* O157:H7 strain Sakai (Yokoyama et al., 2000; Hayashi et al., 2001; Ohnishi et al., 2002; Eppinger et al., 2011). The *stx* insertion sites in the three newly sequenced strains and six *stx*-negative strains from NCBI were identified by screening the DNA sequences of the six *stx* insertion sites from strain Sakai against the chromosomal sequences of each of the nine *E. coli* O157:H7 strains using BLASTn (Johnson et al., 2008). Each of the six *stx* insertion sites in all nine *E. coli* O157:H7 strains was manually checked to determine if these *stx* insertion sites were occupied by any prophages (*stx*-carrying or *stx*-negative) identified by PHASTER.

DNA sequences of three Red recombination genes (*exo*, *bet*, and *gam*) and Red operon repressor gene *cI* were extracted from the whole genome sequence of *E. coli* O157:H7 strain Sakai (Hillyar, 2012). These 4 genes were firstly screened against the prophages at the *stx* insertion sites in the 10 selected strains using BLASTn (Johnson et al., 2008). In addition, we screened all prophages located within in entire genomes of strains Sakai, C1-057 (*stx−*), C1-010 (*stx1−*, *stx2c*+), and C1-067 (*stx1*+, *stx2a*+) for these four genes.

### Identification of Unique Genes Associated With Metabolic Pathways

The whole genome sequences of *E. coli* O157:H7 strain C1-057 (*stx1−*, *stx2-*) and strain Sakai were aligned by DNASTAR MegAlign Pro 14 to identify large specific sequence regions (SSRs) (> 10 kb) that were located in either strain C1-057 or Sakai. Genes that were harbored in SSRs and the sequence regions (10 kb) downstream or upstream of each SSR in either strain C1-057 or Sakai were mapped against the Kyoto Encyclopedia of Genes and Genomes (KEGG) pathway database using the Integrated Microbial Genomes and Microbiomes (accessible at^4^) (Kanehisa et al., 2016; Chen et al., 2018). The unique genes, which were from both SSRs and the sequence regions downstream or upstream of SSRs, with a hit to the genes in the KEGG metabolic pathways were extracted and combined into one FASTA file. Sequences of unique metabolic pathway genes in the FASTA file were mapped against the genomic sequences of the newly sequenced strains, C1-067 (*stx1*+, *stx2*+) and C1-010 (*stx1−*, *stx2c*+), and the six *stx*-negative strains from the NCBI database to identify if any of the metabolic pathway genes were present in any of these strains.

## Results

### General Features of the Three Newly Sequenced *E. coli* O157:H7 Strains

Table 2 shows the general features of the three newly sequenced *E. coli* O157:H7 strains previously isolated from feedlot cattle fecal samples. As already described, each of these three strains had different *stx* profiles. In the current study, the gapless chromosome of the *stx*-negative strain, C1-057 (5.45 Mb), was obtained. The chromosomes of the two *stx*-positive strains, C1-010 (*stx1−*, *stx2c*+) (5.57 Mb) and C1-067 (*stx1*+, *stx2a*+) (5.56 Mb), were composed of 12 and 11 scaffolds, respectively. In addition, the plasmid identification showed that strains C1-057, C1-010, and C1-067 each contained one pO157-like plasmid with sequence similarity of 97% (97.5 kb), 97% (91.9 kb), and 99% (92.7 kb) to the pO157 (92.7 kb) in strain Sakai, respectively. The DNA sequences of these three strains have been deposited to the NCBI (see Table 2 for their NCBI accession number).

**TABLE 2:** General genomic features of the three newly sequenced *Escherichia coli* O157:H7 strains with different *stx* profiles.

	***E. coli* O157:H7 strain C1-057**	***E. coli* O157:H7 strain C1-010**	***E. coli* O157:H7 strain C1-067**
*stx* gene profiles	*stx1−*/s*tx2-*	*stx1−*/*stx2c*	*stx1*/*stx2a*
Chromosome length (bp)	5,452,210	5,571,092	5,564,227
Chromosome contigs	1	12 scaffolds	11 scaffolds
Plasmid no.	1	1	1
Plasmid length (bp)	97,494	91,863	92,720
Coverage	100x	477x	497x
Total coding genes	5,154	5,410	5,378
Total tRNA	101	111	104
NCBI accession number	CP035366	SCKH01000000	RICC01000000

### Phylogenomic Analysis

**FIGURE 1:**
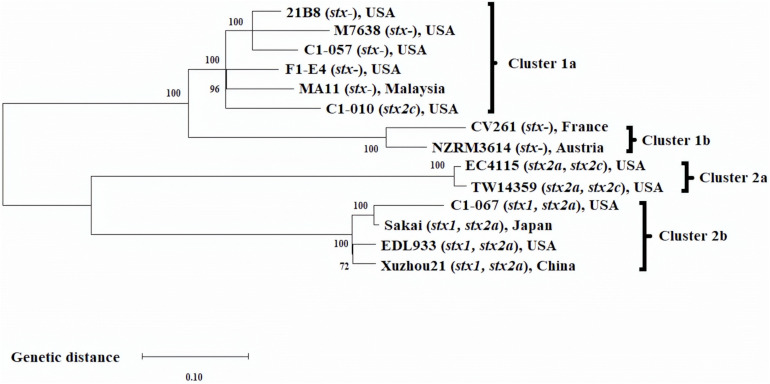
Maximum likelihood phylogenomic tree of 14 *Escherichia coli* O157:H7 strains. SNP matrix was generated from 3,581 SNP with 2,000 bootstraps, and the phylogenomic tree was visualized using MEGA-X.

Single nucleotide polymorphisms analysis was performed to evaluate the phylogenomic relationship among the 14 *E. coli* O157:H7 strains (Figure 1). The optimal length of k-mer was determined as 21 by Kchooser. A total of 3,581 SNP were identified from the 14 strains. Figure 1 shows the ML phylogenomic tree of the 14 *E. coli* O157:H7 strains. Overall, C1-057 (*stx1−*, *stx2-*) and C1-010 (*stx1−*, *stx2c*+), together with the six *stx*-negative *E. coli* O157:H7 strains from the NCBI database, were grouped into Cluster 1, whereas C1-067 (*stx1*+, *stx2c*+) and the five foodborne outbreak strains were grouped into Cluster 2. More specifically, strains C1-057 and C1-010 were clustered in Cluster 1a with three *stx*-negative strains from the United States and one *stx*-negative strain from Malaysia. Strain C1-067 grouped into Cluster 2b with three of the foodborne outbreak strains (Sakai, EDL933, Xuzhou21) had the closest relationship with strain Sakai.

### Prophage Identification

Prophage identification, using PHASTER, was conducted on 10 of the 14 *E. coli* O157:H7 strains. The 10 strains included all the strains that were grouped in Cluster 1 [i.e., C1-010 (*stx1−*, *stx2c*+), C1-057 (*stx−*), and 6 other *stx*-negative strains] and strains C1-067 (*stx1*+, *stx2a*+) and Sakai from Cluster 2b since they had the closest phylogenomic relationship. The results of the identified prophages are shown in Table 3. Overall, 17–32 prophages were identified on the chromosome sequences of the selected *E. coli* O157:H7 strains. These prophages contained genome sizes ranging from 6.3 to 124.9 kb. Strain Sakai is known to have one P4-like prophage Sp2 (12.9 kb) (Asadulghani et al., 2009). The DNA sequence of prophage Sp2 was screened against the other nine selected *E. coli* O157:H7 strains. Six *stx*-negative strains (C1-057, 21B8, M7638, F1 E4, CV261, MA11), strain C1-010 (*stx1−*, *stx2c*+), and strain C1-067 (*stx1*+, *stx2a*+) harbored the identical Sp2 in their genomes. One *stx*-negative strain (NZRM3614) contained a Sp2-like prophage that shared 100% coverage and identity to Sp2 but with an insertion sequence of 1.3 kb.

**TABLE 3:** Different prophage contents, including intact, questionable, and incomplete prophages, predicted from the *Escherichia coli* O157:H7 strains belonging to Cluster 1 and Cluster 2 by PHASTER.

***E. coli* O157:H7 strain**	***stx* profile**	**Number of intact prophages (size)**	**Number of questionable prophages (size)**	**Number of incomplete prophages (size)**
**Cluster 1**				
21B8	N*	10 (14.2–55.4 kb)	4 (10.7–49.6 kb)	3 (7.9–13.8 kb)
M7638	N	11 (14.2–110.5 kb)	4 (10.7–49.6 kb)	3 (7.9–13.8 kb)
C1-057	N	14 (14.2–112.0 kb)	1 (22.4 kb)	3 (6.3–13.8 kb)
F1 E4	N	11 (14.2–55.8 kb)	4 (10.7–49.6 kb)	3 (7.9–13.6 kb)
MA11	N	11 (14.2–114.6 kb)	4 (10.7–49.6 kb)	4 (7.9–16.4 kb)
C1-010	*stx2c*	15 (11.2–101.1 kb)	7 (7.9–50.9 kb)	5 (7.9–41.0 kb)
CV261	N	14 (14.2–124.9 kb)	3 (10.7–49.6 kb)	5 (9.6–29.0 kb)
NZRM3614	N	12 (14.2–110.4 kb)	1 (50.3 kb)	5 (6.4–24.4 kb)
**Cluster 2**				
C1-067	*stx2a*	15 (19.8–64.9 kb)	7 (10.7–43.4 kb)	10 (4.6–26.3 kb)
Sakai	*stx1*, *stx2a*	13 (23.7–103.1 kb)	2 (26.5–41.6 kb)	3 (6.8–13.8 kb)

* N, negative.

Since both strains Sakai and C1-067 (*stx1*+, *stx2a*+) were grouped into Cluster 2b (Figure 1), prophages in strain C1-067 (isolated from feedlot cattle) were compared with the prophages in strain Sakai. The chromosome of *E. coli* O157:H7 strain Sakai was reported by Hayashi et al. (2001) to contain 18 prophages (Sp1–Sp18), and PHASTER identified all these 18 prophages. Sequence alignment between strains Sakai and C1-067 demonstrated that strain C1-067 contained all 18 identical prophages harbored in strain Sakai (data not shown).

Since strains C1-057 (*stx1−*, *stx2-*) and C1-010 (*stx1−*, *stx2c*+) from feedlot cattle were grouped into Cluster 1 (Figure 1), prophages in strain C1-057 were compared with the prophages in strain C1-010. Strains C1-057 and C1-010 shared 10 intact, 1 questionable, and 2 incomplete prophages. Furthermore, strains C1-057 and C1-010 contained unique prophages that were not present in strains C1-010 and C1-057, respectively. Strain C1-057 harbored four unique intact and one unique incomplete prophages, whereas strain C1-010 had four unique intact and one unique questionable prophages. In addition, we found one, five, and three intact, questionable, and incomplete prophages, respectively, in strain C1-010 that shared similar but shorter sequences with the intact prophages in strain C1-057. It is worth noting that the chromosome of strain C1-057 is fully closed, but strain C1-010 was composed of 12 scaffolds. The shorter prophages observed in strain C1-010 compared with those in strain C1-057 might be due to gaps between scaffolds or the deletion of genes from prophages.

Prophages in the newly sequenced strains C1-057 (*stx1−*, *stx2-*) and C1-067 (*stx1*+, *stx2a*+) belonging to Clusters 1 and 2, respectively, were also compared. Strains C1-057 and C1-067 shared three intact, one questionable, and two incomplete identical prophages. Strain C1-057 carried eight intact, one questionable, and one incomplete prophages that were not found in strain C1-067, whereas strain C1-067 contained eight intact, one questionable, and three incomplete unique prophages. In addition, in strain C1-067, two intact, one questionable, and five incomplete prophages shared similar but not identical sequences with the corresponding intact prophages in strain C1-057.

A 14.2 kb intact prophage was only found in each of the strains from Cluster 1 [C1-010 (*stx1−*, *stx2c*+) and seven *stx*-negative strains], but not in the two strains within Cluster 2b (Sakai and C1-067). PHASTER results indicated that this prophage was the most similar to a known phage P4 (11.6 kb, NCBI accession no. NC_001609.1) with 61% query coverage and 97.4% identity. It should be noted that when phage P4 infects *E. coli* cells, it has three known modes of propagation: (1) it can integrate within the host chromosome as a prophage; (2) it can replicate as a plasmid; or (3) it can replicate itself rapidly in its lytic cycle (Briani et al., 2001). Phage P4 contains 19 genes (Briani et al., 2001). The P4-like prophage in the strains from Cluster 1 contained 25 genes, and 10 of which were identical to the genes present in phage P4. It is reasonable to presume that these 10 genes may be involved in one or more of the three modes of propagation.

### Comparison of the *stx* Insertion Sites Occupied With Prophages

The chromosome of the 10 *E. coli* O157:H7 strains selected for prophage identification was further examined for 6 types of previously described *stx* insertion sites (*wrbA*, *sbcB*, *yehV*, *argW*, *yecE*, and *Z2577*). All 10 strains carried all 6 *stx* insertion site genes. As shown in Table 4, three of the *stx* insertion sites of *wrbA*, *yehV*, and *sbcB* were occupied by prophages in 2, 10, and 3 strains, respectively, whereas the other 3 *stx* insertion sites of *argW*, *yecE*, and *Z2577* remained accessible and unoccupied in all 10 strains.

**TABLE 4:** Comparison of prophages at the six *stx* insertion sites in *Escherichia coli* O157:H7 strains Sakai, the three newly sequenced strains, and six *stx*-negative strains from the NCBI database.

***E. coli* O157:H7 strain**	***stx* profile**	***stx* insertion sites**					
		**wrbA**	**sbcB**	**yehV**	**argW**	**yecE**	**Z2577**
**Cluster 1a**							
21B8	*stx–*	–*	–	Sp15-like (R)	–	–	–
M7638	*stx–*	–	–	Sp15-like (R)	–	–	–
C1-057	*stx–*	–	–	Sp15-like (R)	–	–	–
F1 E4	*stx–*	–	–	Sp15-like (R)	–	–	–
MA11	*stx–*	–	–	Sp15-like (R)	–	–	–
C1-010	*stx2c*	–	1717-like-A (RC) (*stx2c*)	Sp15-like (R)	–	–	–
Cluster 1b							
CV261	*stx–*	–	1717-like-B (NA)	Sp15-like (R)	–	–	–
NZRM3614	*stx–*	–	1717-like-C (RC)	Sp15-like (R)	–	–	–
**Cluster 2b**							
Sakai	*stx1*, *stx2a*	Sp5 (RC) (*stx2a*)	–	Sp15 (RC) (*stx1*)	–	–	–
C1-067	*stx1*, *stx2a*	Sp5 (RC) (*stx2a*)	–	Sp15 (RC) (*stx1*)	–	–	–

*No prophage inserted in the insertion site.

*R, The prophage contains three Red recombination genes consisting of *exo*, *bet*, and *gam*.*

*C, The prophage contains the gene *cI* that encodes the Red recombination repressor protein CI.*

*NA, The prophage does not contain neither any of the Red recombination genes nor repressor *cI*.*

Among the 10 selected *E. coli* O157:H7 strains, strains Sakai and C1-067 (*stx1*+, *stx2a*+) belonging to Cluster 2b carried 2 identical *stx*-carrying prophages: 1 *stx1*-carrying prophage at the *yehV stx* insertion site and 1 *stx2a*-carrying prophage at the *wrbA stx* insertion site. All strains from Cluster 1a, except for strain C1-010 (*stx1−*, *stx2c*+), harbored an identical *stx*-negative prophage at the *yehV stx* insertion site. The two strains from Cluster 1b and strain C1-010 carried an identical *stx*-negative prophage at the *yehV stx* insertion site, whereas strain C1-010 contained a *stx2c*-carrying prophage, and the two strains from Cluster 1b contained *stx*-negative prophages with varied DNA sequences at the *sbcB stx* insertion site.

**FIGURE 2:**
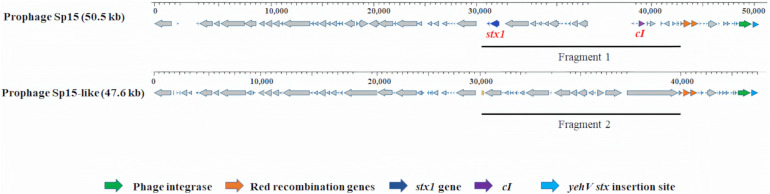
Prophage comparison at *yehV stx* insertion sites in *Escherichia coli* O157:H7 strains used in this analysis. The *stx1*-carrying prophage Sp15 was present in strains Sakai and C1-067 (*stx1* +, *stx2a* +). The *stx*-negative prophage Sp15-like was present in seven *stx*-negative strains and strain C1-010 (*stx1* +, *stx2-*). A 12.8 kb DNA fragment of the *stx1*-carrying prophage Sp15 containing the genes *stx1A* and *stx1B* (fragment 1) and a 10.2 kb DNA fragment that does not contain the *stx* genes of the *stx*-negative prophage Sp15-like (Fragment 2) are presented.

For all 10 selected *E. coli* O157:H7 strains, one specific type of prophage with varied DNA sequences was observed at the *yehV stx* insertion site. The *stx1*-carrying prophage Sp15 (50.5 kb) was present in strains Sakai and C1-067 (*stx1*+, *stx2a*+). An intact *stx*-negative prophage (47.6 kb), named Sp15-like, in all Cluster 1 strains had 72% query coverage and 95% identity to prophage Sp15. We observed a 10.2 kb DNA fragment without the *stx* genes of the prophage Sp15-like in the strains from Cluster 1, whereas a 12.8 kb DNA fragment containing the genes of *stx1A* and *stx1B* of the *stx1*-carrying prophage Sp15 was present in each strain of Sakai and C1-067 (Figure 2).

**FIGURE 3:**
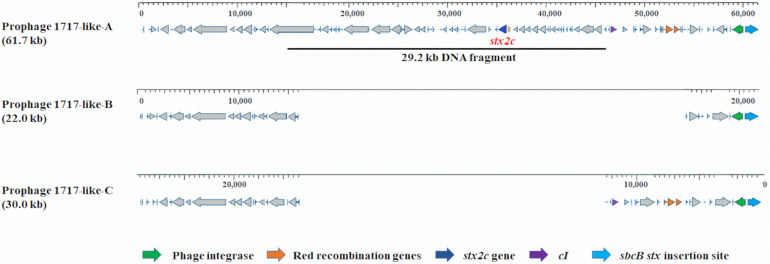
Prophage comparison at *sbcB stx* insertion sites in *Escherichia coli* O157:H7 strains used in this analysis. The *stx2c*-carrying prophages 1717-like-A was present in strain C1-010 (*stx1−*, *stx2c* +). The *stx*-negative prophages 1717-like-B and 1717-like-C were present in strains CV261 and NZRM3614, respectively. A 29.2 kb DNA fragment containing the *stx2c* gene was present in prophage 1717-like-A in strain C1-010, but this DNA fragment was absent in the *stx*-negative prophages 1717-like-B and 1717-like-C in strains CV261 and NZRM3614, respectively.

At the *sbcB stx* insertion site (Figure 3), prophages similar to phage 1717 (62.1 kb, NCBI accession no. FJ188381.1) but with varied sizes and varied query coverages and identities were found in the *stx2c*-carrying strain C1-010 belonging to Cluster 1a and the two *stx*-negative strains from Cluster 1b. A *stx2c*-carrying prophage (61.7 kb), named 1717-like-A, in strain C1-010 had 98% query coverage and 99% identity to phage 1717. A *stx*-negative prophage (22.0 kb, 34% query coverage and 99% identity to phage 1717), named 1717-like-B, and another *stx*-negative prophage (30.0 kb, 47% query coverage and 99% identity to phage 1717), named 1717-like-C, were found in the *stx*-negative strains CV261 and NZRM3614, respectively. Sequence comparisons demonstrated that a 29.2 kb DNA fragment containing the *stx2c* gene was present in prophage 1717-like-A in strain C1-010 belonging to Cluster 1a, but this DNA fragment was absent in the *stx*-negative prophages 1717-like-B and 1717-like-C in strains CV261 and NZRM3614 from Cluster 1b, respectively.

### Comparison of the Red Recombination Genes and Their Repressor Gene *cI*

The prophages located at the 3 *stx* insertion sites in the 10 selected strains were investigated for the presence/absence of Red homologous recombination genes (*exo*, *bet*, and *gam*) and/or their repressor gene *cI* (Table 4). This analysis showed that three types of *stx*-carrying prophages (*stx2a*-carrying prophage Sp5, *stx1*-carrying prophage Sp15, and *stx2c*-carrying prophage 1717-like-A) contained both the three Red recombination genes and their repressor *cI*. Depending on the strain, *stx*-negative prophages had three situations: (1) prophage 1717-like-C in one strain contained both the three Red recombination genes and their repressor *cI*; (2) prophage Sp15-like in eight strains harbored only the three Red recombination genes, but the repressor *cI* was absent; and (3) prophage 1717-like-B in one strain contained neither any of the Red recombination genes nor the repressor *cI*.

The entire genomes of strains Sakai, C1-057 (*stx−*), C1-010 (*stx1−*, *stx2c*+), and C1-067 (*stx1*+, *stx2a*+) were also examined for any other prophages that were not located at the *stx* insertion sites but also harbored the Red recombination gene set. In addition to the prophages at the *stx* insertion sites, only one additional prophage (Sp3, 38.6 kb) carrying *exo* and *bet*, but not *gam* and *cI*, was found in strains Sakai, C1-057, C1-010, and C1-067.

### Genes Involved in Metabolic Pathways Located at SSRs and Sequence Regions Close to SSRs

We found that there were nine SSRs and six SSRs uniquely present in strains Sakai (*stx1*+, *stx2a*+) and C1-057 (*stx−*), respectively. The *stx2a*-carrying *E. coli* O157:H7 strains Sakai and C1-067 carried eight copies of unique metabolic pathway genes, including *sodC*, *modD*, *fepC*, *fecD*, *fbpA*, *dhaM*, *dhaL*, and *dhaK*. On the other hand, the *stx2c*-carrying *E. coli* O157:H7 strain C1-010 and seven *stx*-negative *E. coli* O157:H7 strains contained one copy of unique metabolic pathway gene, *dhaR*. Protein functions of the nine copies of unique metabolic pathway genes are listed in Table 5.

**TABLE 5:** The unique genes involved in metabolic pathways harbored in two *stx2a*-carrying, one *stx2c*-carrying, and seven *stx*-negative *Escherichia coli* O157:H7 strains used in this study.

**Gene symbol**	**Protein**	**Protein function**
*sodC*	Cooper/zinc-superoxide dismutase	Catalyzing the dismutation of the superoxide radical (O_2_^–^) into the hydrogen peroxide (H_2_O_2_). Protecting bacteria from exogenous sources of superoxide
*fepC*	ATP-binding protein	Providing energy to assist the iron uptake system, ferric enterobactin transport system
*fecD*	Ferric citrate transporter permease	Assisting to uptake diferric dicitrate through the cytoplasmic membrane in the iron uptake system, ferric citrate transport system
*fbpA*	Ferric substrate-binding protein	Binding iron substrate that crosses the outer membrane
*dhaM*	Dihydroxyacetone kinase phosphotransferase	Transferring the phosphate to the dihydroxyacetone kinase. Dependent on the sugar phosphotransferase system, an energy transducing system involved in carbohydrate uptake and control of carbon metabolism. Negatively regulating *dhaKLM* operon
*dhaL*	Dihydroxyacetone kinase subunit	Containing ADP as cofactor for the double displacement of phosphate from DhaM to Dha. Positively regulating *dhaKLM* operon
*dhaK*	Dihydroxyacetone kinase subunit	The dihydroxyacetone binding site. Negatively regulating *dhaKLM* operon
*dhaR*	Transcription activator of *dhaKLM* operon	Stimulating transcription of *dhaKLM* operon. Disruption of *dhaR* leads that the *dhaKLM* operon cannot be induced with Dha
*modD*	ModD	Unknown, downstream of the molybdate transporter operon

**FIGURE 4:**
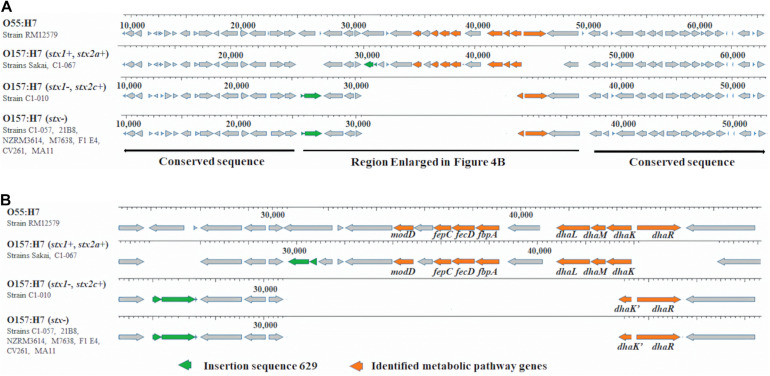
**(A)** Unique metabolic pathway genes and IS629 in 10 *Escherichia coli* O157:H7 strains within a conserved region and metabolic pathway genes in 1 *Escherichia coli* O55:H7 strain within the corresponding conserved region. **(B)** Enlarged region in **(A)**.

In *stx2a*-carrying *E. coli* O157:H7 strains, one copy of unique metabolic pathway gene *sodC* was located at prophage Sp10. The other seven unique metabolic pathway genes in two *stx2a*-carrying strains and one unique metabolic pathway gene in one *stx2c*-carrying and seven *stx*-negative strains were found downstream of an insertion sequence 629 (IS629) within a conserved region among these strains (Figures 4A,B). Previous studies reported that *E. coli* O55:H7 was the ancestor of *stx*-positive and *stx*-negative *E. coli* O157:H7 (Shaikh and Tarr, 2003; Leopold et al., 2009; Byrne et al., 2018). In *E. coli* O55:H7 strain RM12579, we observed a corresponding conserved region that lacked IS629 but harbored a combination of eight unique metabolic pathway genes, including seven genes from *stx2a*-carrying strains and one gene from *stx2c*-carrying and *stx*-negative strains (Figures 4A,B).

## Discussion

In this study, we performed a whole genome analysis of three *E. coli* O157:H7 strains with different *stx* profiles that were previously isolated from feedlot cattle (Carlson et al., 2009). Phylogenomic analysis of the three strains together with 11 other *E. coli* O157:H7 strains showed that these three strains were grouped into different clusters. The *stx*-negative strain C1-057 was grouped with four *stx*-negative strains in Cluster 1a. Strain C1-010, although *stx2c* positive, was grouped with five *stx*-negative strains in Cluster 1a. Strain C1-067, carrying both *stx1* and *stx2a* genes, was grouped into Cluster 2b with strain Sakai (*stx1*+, *stx2a*+). These results suggested that the three *E. coli* O157:H7 strains from feedlot cattle may exhibit different levels of pathogenicity.

Wetzel and LeJeune (2007) reported that *stx*-negative *E. coli* O157:H7 could potentially be converted to *stx*-positive *E. coli* O157:H7 in cattle, swine, and avian production systems. A previous evolutionary model suggested that *stx*-positive *E. coli* O157:H7 could have evolved stepwise from an ancestor *E. coli* O55:H7, following the steps of 1) acquiring *stx2*-carrying prophages, 2) losing the O55 *rfb-gnd* gene, acquiring the O157 *rfb-gnd* gene, and losing the ability to ferment sorbitol, and 3) acquiring *stx1*-carrying prophage (Shaikh and Tarr, 2003; Leopold et al., 2009). However, the exact mechanisms involved in the acquisition and/or loss of the *stx* genes in *E. coli* O157:H7 strains are not fully understood. A greater understanding of the mechanisms involved in the acquisition or loss of *stx* genes could aid in the development of control strategies to reduce or eliminate highly pathogenic *E. coli* O157:H7 strains in cattle.

In addition to acquiring the *stx*-carrying prophage to become *stx*-positive *E. coli* O157:H7 strains, the results of this study suggested a potential alternative mechanism that could result in the acquisition of *stx* genes *via* the Red homologous recombination system. According to Yu et al. (2000), the Red homologous recombination system, which is usually found in lambda prophages, consists of three Red recombination genes, *exo*, *bet*, and *gam*, and the expression of these genes is regulated by their repressor CI (Table 6). The binding of repressor protein CI to the promoter pL in lambda prophages inhibits the expression of the Red recombination genes (Yu et al., 2000). We compared the presence or absence of Red recombination genes and the repressor gene *cI* between *stx*-negative and *stx*-positive prophages (Figures 2, 3). Overall, all *stx*-carrying prophages contained both the three Red recombination genes and their repressor *cI*, whereas the majority of the *stx*-negative prophages at the *stx* insertion sites carried only the three Red recombination genes but not the repressor *cI* gene. Our findings are consistent with the findings of previous studies conducted to examine *stx2*-carrying prophages in *stx*-positive *E. coli* O157:H7 strains (Smith et al., 2012; Tozzoli et al., 2014). These studies also demonstrated that both the three Red recombination genes and their repressor gene *cI* were present in *stx2*-carrying prophages (Smith et al., 2012; Tozzoli et al., 2014).

**TABLE 6:** Genes involved in the Red homologous recombination system in lambda prophages.

**Gene symbol**	**Protein**	**Protein function**
*exo*	Exo	Cleaving the 5′-end of dsDNA and form 3′-end overhangs
*bet*	Bet	Protecting the ssDNA generated by Exo and promoting its annealing to a complementary ssDNA target in *E. coli* cells
*gam*	Gam	Preventing the endogenous nucleases (RecBCD and SbcCD) from digesting foreign linear DNA that is introduced into the *E. coli* cells
*cI*	Repressor CI	Inhibiting the expression of genes *exo*, *bet*, and *gam* in prophage

As previously mentioned, the presence of repressor CI in *stx*-carrying prophages could inhibit the activity of Red homologous recombination, and consequently, the stability of the *stx* genes in *stx*-positive *E. coli* O157:H7 could be increased. On the other hand, the majority of the *stx*-negative prophages at the *stx* insertion sites in this study carried only the three Red recombination genes, but their repressor *cI* was absent. In the absence of the repressor *cI* in *stx*-negative prophages, the consistent expression of the Red recombination genes may result in more frequent gene exchange, potentially contributing to the acquisition of *stx* genes. Overall, our results indicate that the presence or absence of repressor *cI* might result in different levels of activity of Red recombination in *stx*-positive and *stx*-negative prophages. For the *stx*-carrying prophages containing both the Red recombination genes and their repressor *cI*, the potential of losing *stx* genes is relatively low due to the inhibition of the activity of Red homologous recombination by their repressor CI. However, for the *stx*-negative prophages containing only Red recombination genes but not repressor *cI*, the potential of acquiring *stx* genes is relatively high due to the consistent expression of Red recombination genes, resulting in frequent gene exchanges. To our knowledge, this is the first report to suggest the possible functions of Red recombination genes and their repressor relating to the stability and acquisition of *stx* genes. Further studies are required to verify this proposed function associated with Red recombination genes and their repressor *cI* in more *stx*-negative and *stx*-positive *E. coli* O157:H7 strains under laboratory conditions and in the cattle environment.

The presence or absence of *stx* genes may also result in changes in other genotypic traits between *stx*-positive and *stx*-negative *E. coli* O157:H7, such as antibiotic resistance genes (Garcia-Aljaro et al., 2009; Colavecchio et al., 2017). In this study, we identified eight copies of unique metabolic pathway genes in two *stx2a*-carrying *E. coli* O157:H7 strains and one copy of a unique metabolic pathway gene in one *stx2c*-carrying and seven *stx*-negative *E. coli* O157:H7 strains. Among these nine metabolic pathway genes, gene *sodC* might contribute to the increased survival of *stx*-positive *E. coli* O157:H7 in complex microbial communities (Battistoni, 2003). Genes *fepC*, *fecD*, and *fbpA* could potentially be associated with improved iron uptake ability (Staudenmaier et al., 1989; Chen et al., 1993; Raymond et al., 2003). Genes *dhaK*, *dhaL*, *dhaM*, and *dhaR* are associated with glycerol metabolism (Bächler et al., 2005). The presence of more unique metabolic pathway genes in *stx*-positive (*stx1*+, *stx2a*+) strains may potentially lead to an increase in their competitiveness in complex environments, such as feedlot cattle.

The *sodC* gene encodes copper/zinc-superoxide dismutase (Cu–Zn SOD) that catalyzes the dismutation of the superoxide radical (O_2_^–^) into hydrogen peroxide (H_2_O_2_) (Fridovich, 1981). The *sodC* is considered a virulence gene since the Cu–Zn SOD protects bacteria from exogenous sources of superoxide. A previous study indicated that Cu–Zn SOD protected *E. coli* strains from superoxide generated by macrophages and enhanced bacterial survival (Battistoni, 2003). We found one copy of *sodC* harbored on the chromosomes of all 10 strains used in this analysis. However, an additional copy of *sodC* harbored in the Sp10 prophage was found only in Sakai and C1-067 (*stx1*+, *stx2a*+) but was absent in strains C1-057 (*stx1−*, *stx2-*), C1-010 (*stx1−*, *stx2c*+), and the six *stx*-negative strains from the NCBI database. The second copy of *sodC* may provide additional protection from the toxicity of superoxide radical. Thus, the *stx*-positive strains Sakai and C1-067 may be more resistant to exogenous superoxide radical than strains C1-057, C1-010, and the six *stx*-negative strains from the NCBI database.

The *fepC*, *fecD*, and *fbpA* genes encode proteins that are involved in three separate iron uptake systems, respectively. The *fepC*, from the ferric enterobactin transport system FepABCDG, encodes an ATP-binding protein transport that provides energy to assist iron uptake from the environment (Raymond et al., 2003). The *fecD*, from the ferric citrate transport system FecABCDE, encodes a ferric citrate transporter permease that assists to uptake diferric dicitrate through the cytoplasmic membrane (Staudenmaier et al., 1989). The *fbpA*, from the iron uptake gene cluster *fbpABC*, encodes ferric substrate-binding protein (Chen et al., 1993). Although this transposable element (TE) did not harbor any of the intact iron uptake gene cluster, the presence of genes *fepC*, *fecD*, and *fbpA* in strains Sakai and C1-067 (*stx1*+, *stx2a*+) can potentially increase iron uptake ability compared with strains C1-057 (*stx1−*, *stx2-*), C1-010 (*stx1−*, *stx2c*+), and the six *stx*-negative strains from the NCBI database.

*Escherichia coli* can metabolize glycerol *via* two microaerobic pathways: the dihydroxyacetone kinase (DAK) pathway and the glycerol pathway (Durnin et al., 2009). DhaK, DhaL, and DhaM, encoded by the *dha* operon, are proteins to phosphorylate DHA in the DAK pathway. The *dha* operon is controlled by DhaR, a transcriptional activator (Bächler et al., 2005). All four proteins are essential for the DAK pathway. Based on the O serotype and *stx*-profiles, we observed four *dha* operons (Figure 4B): (1) *E. coli* O55:H7 strain RM12579 harbored an intact *dha* operon consisting of *dhaK*, *dhaL*, *dhaM*, and *dhaR*; (2) *E. coli* O157:H7 *stx*-positive strains Sakai and C1-067 (*stx1*+, *stx2a*+) harbored a *dha* operon containing *dhaK*, *dhaL*, and *dhaM*, but not *dhaR*; (3) *E. coli* O157:H7 *stx*-positive strain C1-010 (*stx1−*, *stx2c*+) harbored another *dha* operon comprising only *dhaR* but not *dhaK*, *dhaL*, or *dhaM*; and (4) seven *E. coli* O157:H7 *stx*-negative strains harbored the same disrupted *dha* operon as strain C1-010. Additionally, both two types of disruptions of *dha* systems as shown in strains C1-067 and C1-010 inactivated the metabolism of glycerol *via* the DAK pathway. Durnin et al. (2009) reported that the deletion of *dhaK* led to a reduction in the efficacy of glycerol utilization by approximately 50%, resulting in a reduction in cell growth rate by 70%, in a minimal medium supplemented with glycerol as the sole carbon source. Another study also indicated that adding three levels of crude glycerin (0, 4, and 8%) in corn silage diets decreased the prevalence levels of *E. coli* O157 to 4.4, 3.2, and 1.8% in fecal samples from growing cattle, respectively (*P* < 0.01) (Aperce et al., 2013). Our results provided evidence that the disruption of the DAK pathway in both *stx*-positive and *stx*-negative *E. coli* O157:H7 strains from this study might contribute to a decrease in the rate of glycerol metabolism. Therefore, the disruption of the DAK pathways can potentially decrease the competitiveness and survival of *E. coli* O157:H7 in growing cattle fed diets that include crude glycerin. However, the addition of glycerol as a supplement may result in varying effects on the growth and activity of rumen bacteria. For example, high levels of glycerol (72–108 g/kg dry matter) in feed decreased (*P* < 0.05) the DNA concentrations of fiber-fermenting bacteria *Butyrivibrio fibrisolvens* and *Selenomonas ruminantium* in rumen, potentially resulting in the reduction of fiber digestibility (AbuGhazaleh et al., 2011). However, glycerol had no effect on fiber-fermenting bacteria *Ruminococcus albus* and starch-digesting bacteria *Succinivibrio dextrinosolvens* (El-Nor et al., 2010; AbuGhazaleh et al., 2011). In addition, the numbers of Enterobacteriaceae and *Lactobacillus* in rumen were not affected when cattle were fed oral rehydration solution containing glycerol (Omazic et al., 2013). The addition of glycerol in animal feed to manipulate rumen microflora for reducing pathogenic bacteria while at the same time retaining beneficial microflora could be complicated and, therefore, requires careful consideration and further investigation.

Among the metabolic pathway genes discussed above, seven unique metabolic pathway genes in the two *stx2a*-carrying strains and one in the one *stx2c*-carrying and seven *stx*-negative strains were found downstream of an IS629 within a conserved region (Figure 4A). Previous studies reported that *E. coli* O157:H7 could have evolved from an ancestor *E. coli* O55:H7 (Shaikh and Tarr, 2003; Leopold et al., 2009; Byrne et al., 2018). Sequence alignment demonstrated that a corresponding conserved region was also observed in the *E. coli* O55:H7 strain. Unlike the 10 *E. coli* O157:H7 strains, the corresponding conserved region in the *E. coli* O55:H7 strain lacked IS629, but it contained all eight metabolic pathway genes, including seven from *stx2a*-carrying *E. coli* O157:H7 strains and one from *stx2c*-carrying and *stx*-negative *E. coli* O157:H7 strains. Researchers used to believe that excision of IS elements rarely occurred in bacteria due to no regeneration of donor DNA by end-joining activity after IS excision (Synder and Champness, 2003). However, a study reported that excision of IS629 could occur frequently in *E. coli* O157, causing the diversity of genomic structure in *E. coli* O157 (Kusumoto et al., 2011). IS629 has been reported to be associated with a variety of genomic arrangements, including gene deletions, inversions, and duplications in *Shigella flexneri* (Wei et al., 2003) and *E. coli* O157:H7 (Iguchi et al., 2006; Ooka et al., 2009; Kusumoto et al., 2011). Our results indicated that insertion of IS629 may lead to the different presence of unique metabolic pathway genes between *E. coli* O157:H7 strains with different *stx* profiles. Additionally, for the *stx2c*-carrying and *stx*-negative *E. coli* O157:H7 strains, the fact that they shared the same unique metabolic pathway genes within the conserved region and they were grouped into the same phylogenomic cluster demonstrated that they had a closer genomic relationship, indicating that they may share more closely related genomic evolutionary pathways. Gene exchange between *stx*-negative and *stx2c*-carrying *E. coli* O157:H7 was more likely to occur.

In summary, our study firstly reported the potential functions of Red recombination genes and their repressor *cI* associating with the maintenance of *stx* genes in *stx*-carrying prophages and the acquisition of *stx* genes in *stx*-negative prophages. The presence of repressor CI in *stx*-carrying prophages could inhibit the activity of Red homologous recombination. Consequently, the absence of repressor *cI* in *stx*-negative prophages at the *stx* insertion sites could potentially lead to the consistent expression of Red recombination genes. As a result, gene exchange might occur more frequently, potentially contributing to the acquisition of *stx* genes in the *stx*-negative prophages at the *stx* insertion sites. Additionally, *stx2a*-carrying *E. coli* O157:H7 contained more copies of unique metabolic pathway genes than *stx2c*-carrying and *stx*-negative *E. coli* O157:H7. The presence of more unique metabolic pathway genes in *stx2a*-carrying *E. coli* O157:H7 may potentially lead to an increase in their competitiveness and survival in complex environments, such as feedlot cattle. Furthermore, within a conserved region, we observed seven unique metabolic pathway genes in *stx2a*-carrying *E. coli* O157:H7 and one unique metabolic pathway gene in *stx2c*-carrying and *stx*-negative *E. coli* O157:H7 strains downstream of an IS629. The difference in the presence of metabolic pathway genes of *E. coli* O157:H7 with different *stx* profiles may be associated with insertion of IS629 from their ancestor *E. coli* O55:H7. The fact that *stx2c*-carrying and *stx*-negative *E. coli* O157:H7 strains were grouped into the same cluster and they shared unique metabolic pathway genes within the conserved region suggested that *stx2c*-carrying and *stx*-negative *E. coli* O157:H7 had closely related evolutionary pathways. Gene exchange between *stx*-negative and *stx2c*-carrying *E. coli* O157:H7 was more likely to occur. Our comparative genomic analysis also revealed that *E. coli* O157:H7, regardless of carrying *stx* genes or not, did not contain a functional *dha* operon. This result indicated that neither *stx*-positive nor *stx*-negative *E. coli* O157:H7 could not utilize glycerol *via* the DAK pathway. Adding glycerol as a feed supplement may be the potential strategy to reduce pathogenic *E. coli* O157:H7 in livestock and downstream food production processing environments.

## Data Availability Statement

The datasets presented in this study can be found in online repositories. The names of the repository/repositories and accession number(s) can be found below: https://www.ncbi.nlm. nih.gov/, CP035366.1, https://www.ncbi.nlm.nih.gov/, CP0353 67.1, https://www.ncbi.nlm.nih.gov/, NZ_SCKH00000000, https://www.ncbi.nlm.nih.gov/, RICC01000000.

## Author Contributions

MJ conceived the presented idea and carried out the experiment and wrote the manuscript with the support from HY. HY helped supervise the project. All authors provided critical feedback and contributed to the final version of the manuscript. All authors contributed to the article and approved the submitted version.

## Conflict of Interest

The authors declare that the research was conducted in the absence of any commercial or financial relationships that could be construed as a potential conflict of interest.

## Footnotes

1 https://sourceforge.net/projects/ksnp/files/

2 www.megasoftware.net

3 www.phaster.ca.

4 https://img.jgi.doe.gov/
